# The role of protein modifications in senescence of freeze-dried *Acetobacter senegalensis* during storage

**DOI:** 10.1186/1475-2859-13-26

**Published:** 2014-02-19

**Authors:** Rasoul Shafiei, Raziyeh Zarmehrkhorshid, Azeddine Bentaib, Manoochehr Babanezhad, Pierre Leprince, Frank Delvigne, Philippe Thonart

**Affiliations:** 1 Walloon Center of Industrial Biology, University of Liège, Liège, Belgium; 2 Department of Biology, Faculty of Sciences, University of Isfahan, Isfahan, Iran; 3 GIGA–Neuroscience, University of Liège, Liège, Belgium; 4 Department of Statistics, Faculty of Sciences, Golestan University, Gorgan, Iran; 5 Bio-industry Unit, Gembloux Agro-Bio Tech, Gembloux, University of Liège, Liège, Belgium

**Keywords:** Starter, Acetic acid fermentation, Oxidative stress, Carbonylation, AGEs, Acetobacter, 2D-DiGE, VBNC, Protein

## Abstract

**Background:**

Loss of viability is one of the most important problems during starter culture production. Previous research has mostly focused on the production process of bacterial starters, but there are few studies about cellular protein deterioration causing cell defectiveness during storage. In the present study, we investigated the influence of storage temperature (−21, 4, 35°C) on the cellular protein modifications which may contribute to the senescence of freeze-dried *Acetobacter senegalensis*.

**Results:**

Heterogeneous populations composed of culturable cells, viable but non-culturable cells (VBNC) and dead cells were generated when freeze-dried cells were kept at −21 and 4°C for 12 months whereas higher storage temperature (35°C) mainly caused death of the cells. The analysis of stored cell proteome by 2D-DiGE demonstrated a modified pattern of protein profile for cell kept at 4 and 35°C due to the formation of protein spot trains and shift of Isoelectric point (pI). Quantification of carbonylated protein by ELISA showed that the cells stored at 4 and 35°C had higher carbonylated protein contents than fresh cells. 2D-DiGE followed by Western blotting also confirmed the carbonylation of cellular proteins involved in translation process and energy generation. The auto-fluorescent feature of cells kept at 35°C increased significantly which may be an indication of protein glycation during storage. In addition, the percentage of cellular unsaturated fatty acid and the solubility of cellular proteins decreased upon storage of cells at higher temperature suggesting that peroxidation of fatty acids and possibly protein lipidation and oxidation occurred.

**Conclusions:**

High storage temperature induces some deteriorative reactions such as protein oxidation, lipidation and glycation which may cause further protein modifications like pI-shift, and protein insolubility. These modifications can partly account for the changes in cell viability. It can also be deduced that even moderate carbonylation of some critical cellular proteins (like ribosomal proteins) may lead to VBNC formation or death of freeze-dried bacteria. Moreover, it seems that other mechanisms of biomolecule deterioration preceding protein carbonylation lead to VBNC formation under very low storage temperature.

## Introduction

A starter culture may be defined as a preparation containing large numbers of viable and culturable microorganisms, which may accelerate a favorable fermentation process [1]. Vinegar technology is one of those fermentation technologies which still suffer from the lack of cost-effective starter cultures [2] due to the genetic instability of acetic acid bacteria during preservation [3] and the susceptibility of cells to downstream processes [4,5].

The viable cells which are used as vinegar starter must be able to tolerate acetic acid and metabolize ethanol efficiently under aggressive conditions of acetic acid fermentation [6]. The availability of appropriate vinegar starter cultures is desirable when a new fermentation run has to be started or when it needs to be restarted due to a sudden interruption of fermentation [2]. In recent years, *Acetobacter senegalensis*, a thermo-tolerant bacterium, has been used for starter production and vinegar production in a pilot plant scale acetifier [4,7]. Use of this strain in vinegar industry can decrease the cooling cost of bioreactors especially in tropical regions. In addition, since this strain can remain viable and active in a wide range of temperature during acetous fermentation [8], any fluctuation of fermentation temperature can be tolerated readily.

There are different industrial techniques to preserve microbial starters; however despite being an expensive and long process, freeze-drying is one of the most convenient and applicable methods compared to other drying methods [9]. However, as with other drying methods, loss of viability is one of the main problems during freezing and drying processes as well as storage period [4].

Intrinsic and environmental factors have considerable influence on the viability of microbial starters [9]. Generally, the survival rate of gram-positive bacteria immediately after freeze-drying tends to be higher than that of gram-negative bacteria [10]. In addition, pre-adaptation of cells in different culture media, usage of protectants and storage conditions affect bacterial viability significantly [5,11]. In this context, there are evidences that damage to DNA, cell wall and cell membrane of bacterial cells under different storage conditions occur [12,13], and it is now well accepted that membrane lipid peroxidation and change in the degree of un-saturated lipids strongly affect the survival of bacteria [7,9,13]. Furthermore, damage to ribosomes and their functions are speculated as the primary reasons of cell viability loss [11].

Cellular proteins are also subjected to deteriorating reactions during downstream processes. They are very susceptible to various modifications due to the range of functional groups displayed by amino acids [14]. Damage to proteins such as oxidation during preservation process and storage period has been studied in animal cells, plant cells, seeds as well as in therapeutic products [15-19]. Oxidative changes of cellular proteins can lead to diverse detrimental consequences in structure and function of proteins such as inhibition of enzymatic activities, polymerization, loss of solubility, increased susceptibility to aggregation and proteolysis [14,20].

Although several protein oxidative modifications exist, most oxidized proteins exhibit carbonyl groups (aldehydes and ketones) [20]. However, compared to other oxidative modifications, carbonylation is relatively difficult to be induced and in contrast to, for example, methionine sulfoxide and cysteine disulfide bond formation, carbonylation is an irreversible oxidative process [21,22]. Carbonyl groups are introduced into the proteins through a variety of oxidative pathways: (I) a decline in the antioxidant defense system, (II) an increased production of reactive oxygen species (ROS), (III) a diminished capacity for removal of oxidized proteins, or (IV) an increased susceptibility of proteins to oxidative attack [23]. In addition, carbonyl derivatives are formed by a direct metal catalyzed oxidative (MCO) attack on the amino-acid side chains of proline, arginine, lysine, and threonine. Furthermore, carbonyl derivatives on lysine, cysteine, and histidine can be formed by secondary reactions with reactive carbonyl compounds on carbohydrates, lipids, and Advanced Glycation/lipoxidation End Products (AGEs) [23].

Advanced Glycation End products (AGEs) and AGE pigments (known also as lipofuscin) are created through non-enzymatic reactions (the Maillard reaction) between reducing sugars and free amino groups of proteins, lipids, or nucleic acids. These biomolecules contain transition metals such as iron, zinc, manganese and copper. These metals can cause a redox-active surface that can catalyze reactive releasing process [24]. AGEs alter the structure of proteins and compromise their functions. The rate of accumulation of AGEs in proteins may be viewed as an index of the rate of damage to other biomolecules, including lipids, glycol-conjugates and DNA [25]. A decreased enzymatic activity through oxidative reaction of proteins and Maillard reaction products has been demonstrated in plant seeds during storage [26,27]. However, there are few studies which address the protein modifications in bacterial cells during desiccation [17].

Despite the progress made so far in the context of bacterial starter production, to our knowledge there are only few studies on the cellular protein modifications occurring during storage of bacterial starters. The aim of this study was to investigate the influences of storage temperature on cellular viability and senescence of freeze-dried *A. senegalensis*. Specifically, the occurrence of modifications in protein content and properties affecting the survival of stored bacteria was studied. In this regard, three storage temperatures (−21, 4, and 35°C) were chosen and freeze-dried cells were stored for 12 months in sealed vials at the three mentioned temperatures. Then, the oxidative damage and its subsequent effects on some cellular protein properties were analyzed. In addition, the consequence of protein oxidation on some other cellular features such as AGEs formation or cell respiration was studied.

## Results and discussion

### Heterogeneous populations are formed during storage of freeze-dried cells

Prolonged stability of freeze-dried starters is crucial for fermentation industry. Studies from our laboratory and others have shown that culturability and the viability of freeze-dried starters are dependent upon freeze-drying process as well as storage conditions [4,9,11,13,28-30]. We have also previously shown that storage temperature affect the viability and culturability of freeze-dried starter, and that even at low storage temperature (−21, 4°C), cells enter into VBNC state [4].

Culturability of cells was determined merely after freeze-drying (as control) and also after 12 months of storage at different temperatures. Cell culturability was affected considerably by storage temperature. As shown in Table 1, while the change in culturability at −21°C was limited, it decreased significantly at 4°C in comparison to −21°C. Additionally, the culturability of cells disappeared at 35°C. It is now well accepted that for long term storage, inactivation of the dried starter cultures extensively depends on the storage conditions [11]. Since the vials containing the freeze-dried samples were completely sealed, the moisture content did not change significantly (Table 1). In addition, they were kept in the dark, and originated from the same fermentation and freeze-drying batches; thus we can assume that the only affecting factor was storage temperature.

**Table 1:** Culturable cells and moisture content of freeze-dried cells kept at various temperatures for 12 months

	**After freeze-drying process**	**Storage temperature**		
		**−21°C**	**+4°C**	**+35°C**
**Direct count (cfu/g)**	5.96E + 11	6.01E + 11	6.21E + 11	5.89E + 11
**cfu/g on GY agar**	1.90E + 11	1.11E + 11	1.03E + 10	<E + 2
**Dry matter (%)**^**1**^	91.60 ± 1.64	91.7 ± 2.14	92.53 ± 1.18	93.29 ± 2.34

^1^Values are presented as mean ± SD.

**Figure 1:**
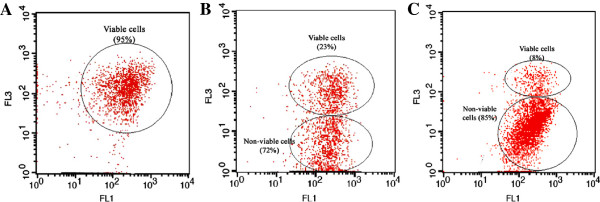
**Flow-cytometric analysis of cellular dehydrogenases of freeze-dried *A. senegalensis *cells stored at −21°C (A), 4°C (B) and 35°C (C) for 12 months.** Thiazole orange (TO) was used to stain all the cells (viable and non-viable cells). CTC is reduced by active cellular dehydrogenases, and the emitted light is absorbed on FL3. The activity of dehydrogenases which determined by CTC reduction was considered as a sign of viability.

Measurement of total dehydrogenase activity (indicative of cell respiration) revealed that nearly all of the cell population (95%) kept at −21°C for 12 months was able to reduce CTC in the presence of glucose-phosphate buffer (Figure 1A). The small percentage of cells which were not able to reduce CTC were presumably dead cells or cells that could not be active under mentioned conditions.

Storing of freeze-dried cells at 4°C for 12 months resulted in a heterogeneous population of bacteria according to CTC reduction ability (Figure 1B). This may indicate that some cellular enzymes involved in respiration system such as dehydrogenases were subjected to detrimental conditions which disabled the respiration system. However, about 23% of cells were able to carry out that reaction completely, resulting in high absorbance on FL3. It has been already shown that dehydrogenases are especially heat sensitive [31]. In our previous study, it was shown that storage of freeze-dried *A. senegalensis* at 4°C for nine months did not change the cell envelope integrity considerably [4], therefore the changes in dehydrogenase activity during storage at 4°C may not be due to the leakage of cellular components or entrance of liquids into the cells. In contrast, it could be due to direct inactivation of enzymes.

**Figure 2:**
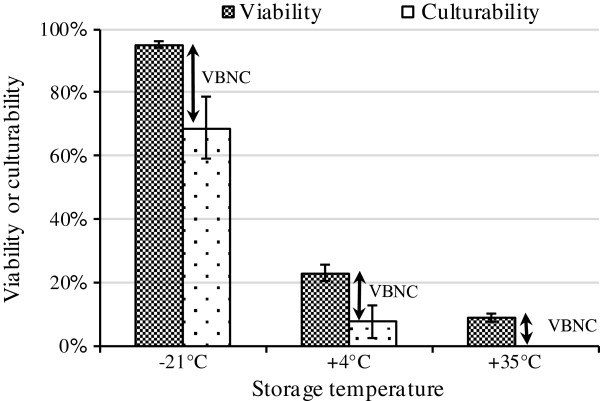
**The influence of storage temperature on VBNC formation in freeze-dried *A. senegalensis *cells after 12 months of storage.** Experiments were performed at least in three independent replicates. Error bar shows the standard deviation.

At 35°C, 85% of cells lost the capability of reducing CTC (Figure 1C). These cells can be considered as dead cells or cells needing some complementary components to perform respiration. Furthermore, our results indicate that although low temperature could decrease the proportion of non-viability and non-culturability, a fraction of cells entered into VBNC state at very low storage temperatures (i.e., -21°C) as indicated by summarizing the data in Figure 2. These results are in line with research showing that VBNC formation is also induced during preservation [32,33]. One possible explanation for entry of bacterial cells into VBNC state during storage period is irreparable damage to key structural or functional components (such as ribosomes and DNA) of cells which are completely necessary for multiplication [11,34,35].

### Formation of fluorescent compounds is enhanced during storage at high temperature

**Figure 3:**
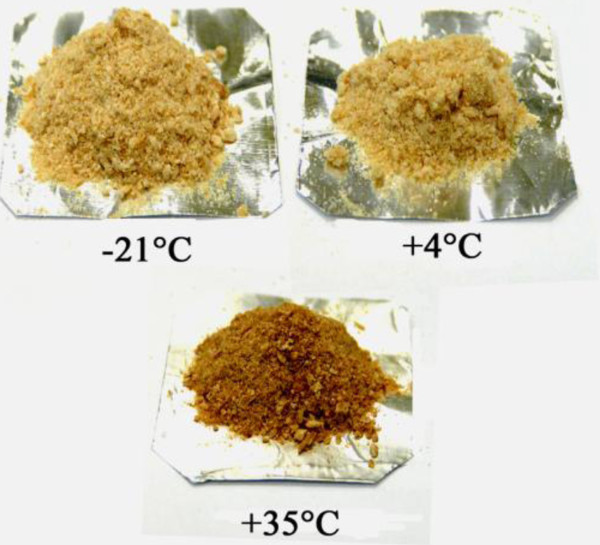
**The appearance of freeze-dried *A. senegalensis *kept at different storage temperatures for 12 months.** The color of freeze-dried cells preserved at 35°C changed to dark brown while the color of freeze-dried cells preserved at −21 and 4°C did not change.

One of the primary diagnostic criteria for development of a browning reaction in a product is the development of dark color in product. As shown in Figure 3, a browning reaction occurred in cells kept at 35°C that darkened after storage for 12 months, whereas the cells stored at lower temperature showed no visible change in color. Non-enzymatic browning may result from Maillard and Amadori reactions starting from a condensation reaction between reducing sugars and amino groups. These reactions are known to produce carbonyl intermediates which react with neighboring amino groups [36]. Protein glycation is a process in which reducing sugars interact with primary amines on the side chains of Lysine and Arginine, resulting in a chemical sequence of reactions known as “Amadori rearrangement” which leads to the formation of Amadori modified proteins (AMPs). AMPs can be further developed, in an oxidation-dependent manner, to form irreversible, highly stable compounds known as Advanced Glycation End-products (AGEs) [37].

**Figure 4:**
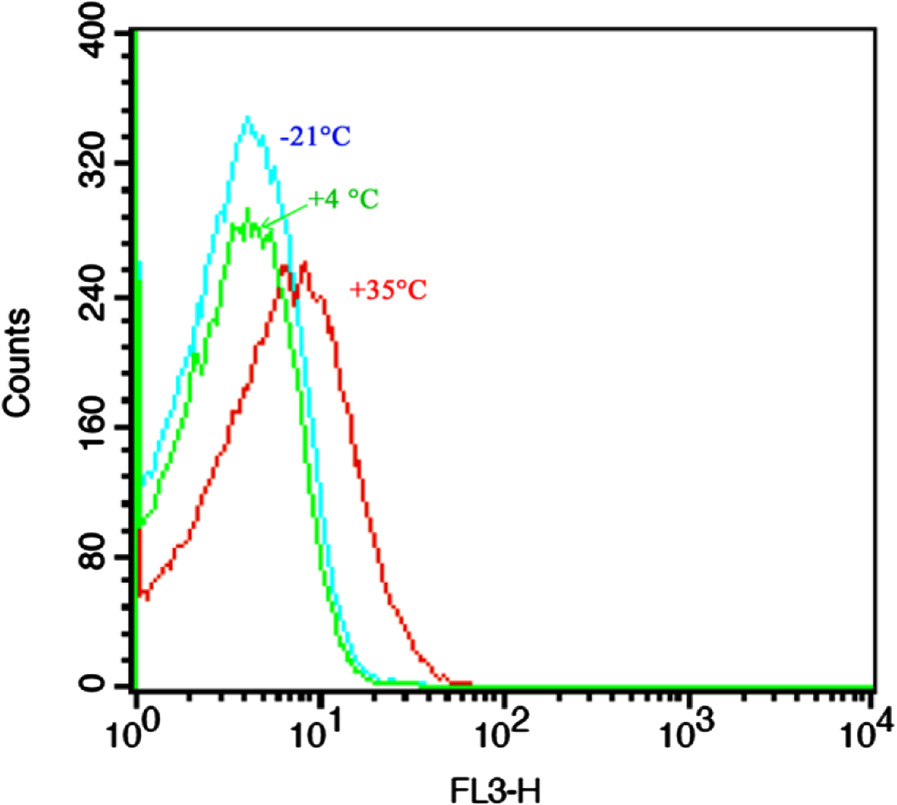
**Auto fluorescence feature of freeze-dried *A. senegalensis *kept at different storage temperatures for 12 months.** Light emitted from cells was collected and appeared on FL3. A significant difference (*p* < 0.05) was observed between the light intensity mean of cells kept at 35°C and the light intensity mean of cells stored at −21 or 4°C.

In order to assess the development of AGEs in cells kept at different storage temperatures, the autofluorescent feature of cells were assessed by flow-cytometric technique. As Figure 4 shows, illumination of cells with blue laser light (488 nm), induced light emission which appeared on FL3. Since the *Kolmogorov*–*Smirnov* test showed that the signal intensity followed a normal distribution, *Duncan’s* multiple range test was used to compare the mean of the three populations. A significant difference in mean and median light intensity was observed between the cells kept at 35°C and −21°C or 4°C. However, there is no significant difference in light intensity between the cells kept at 4 and −21°C. Statistical analysis revealed that about 42% of cells kept at −21 and 4°C had overlap whereas there is no significant overlap between the cells kept at 35°C with the cells kept at lower storage temperature.

Recently, the evaluation of fluorescent compounds generated by the Amadori rearrangement product has become a routine practice. Besides its use in food quality control, fluorescence measurement is also employed to evaluate Maillard reactions causing AGEs generation under physiological conditions, and also to assess AGEs development under pathological conditions [38]. The results obtained in this study are in agreements with those of Kurtmann and coworkers. They showed that browning of freeze-dried *Lactobacillus acidophilus* during storage under relatively mild conditions resulted in various types of non-enzymatic browning reactions including carbonyl-protein (or carbonyl-DNA) interactions and carbohydrate condensation/polymerization [28]. Furthermore, in our study, since the cells were not washed prior to freeze-drying (to avoid possible damage to the cells by low osmotic buffers) [4,5], residual reducing sugar (glucose) and proteins or even DNA or RNA released by cell lysis during fermentation or freeze-drying can enhance the non-enzymatic browning reactions [39].

It is believed that formulation strategies during freeze-drying process have great influence on the stability and viability of bacteria. Addition of compounds containing carbonyl groups has been suggested to be a cause of mortality occurring during storage of dried microorganisms [40].

Form these results, it can be deduced that some temperature-dependent reactions induce the formation of fluorescent compounds in the stored cells. These fluorescent compounds can be a sign of protein glycation.

### The proteome is markedly affected in freeze-dried cells stored at high temperature

To study the molecular mechanisms associated with the loss of viability and culturability during storage, a differential proteomic analysis approach was used. First, we determined the distribution pattern of the cellular proteome by two-dimensional difference gel electrophoresis (2D-DiGE) for cell kept at different temperatures. In the second part, the extent of protein carbonylation was studied using ELISA test and 2D-DiGE followed by immunoblotting (Western blotting).

**Figure 5:**
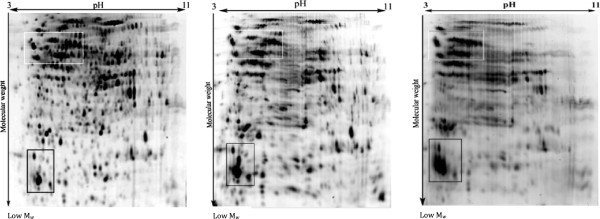
**2D-DiGE of *A. senegalensis *preserved at −21 (left), 4 (middle) and 35°C (right).** The proteins were pre-stained with Cy3 before separation. As it is evident, slight and moderate streaking and trains of spots were formed in the proteome pattern of cells kept at 4 and 35°C. The density of high molecular weight proteins at the basic side of the gel decreased at 35°C. The selected sections (white and black rectangles) show clearly the differences between the patterns of proteins.

**Figure 6:**
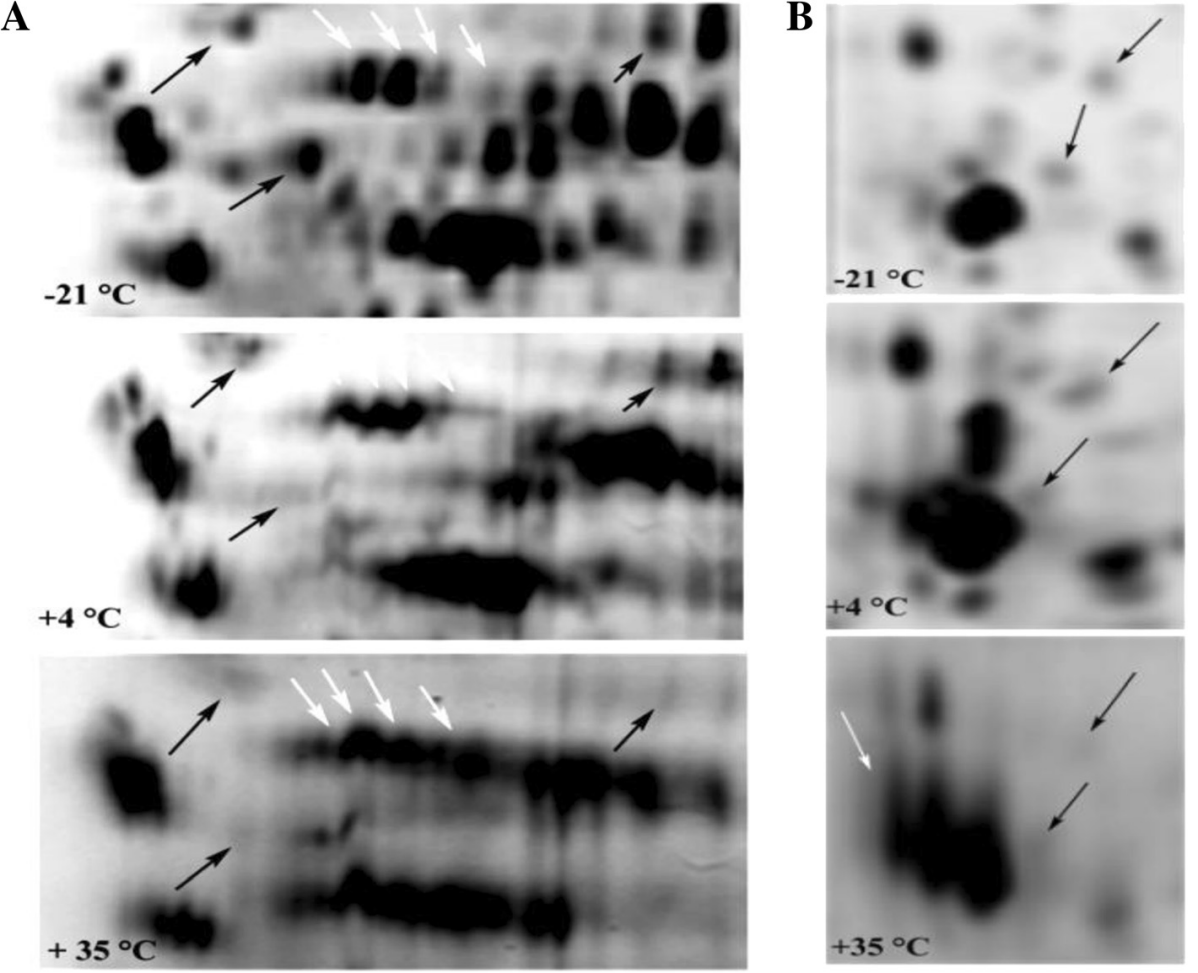
**Comparison of selected 2D-profiles of proteins extracted from cells stored at different temperatures. A** and **B** are the magnified sections (white and black rectangles) in Figure 5, respectively. The black arrows show the protein spots which exhibited a decrease in density during storage at 4 or 35°C. The white arrows show the intact protein spots (−21°C) and the trains of protein spots formed during storage at 4 or 35°C.

The electrophoretic patterns of cellular proteins revealed major changes in protein profile of freeze-dried *A. senegalensis* kept at different storage temperatures (Figure 5). Triplicate samples of cells stored at each temperature were run on different gels, and representative gels are exhibited in Figures 5 and 6. The pattern of obtained spots was reproducible for each condition and includes many proteins appearing as single spots (for the cells kept at −21 and 4°C); however the majority of proteins appeared as trains of spots (white rectangles in Figure 5) or vertical streaks (the black rectangles in Figure 5) for samples kept at 35°C. These changed patterns made it difficult to distinguish unambiguously each protein from neighboring proteins. Different reasons can possibly explain the appearance of streaking in 2D-DiGE: inadequate isoelectric focusing and/or (bio) chemical modification of proteins. Contamination with salts, DNA, polysaccharides and lipids during preparation of protein samples can be a cause of streaking. Since the same standard procedure was applied to all the samples using high grade reagents, it seems that streaking cannot arise from the sample preparation procedures. In contrast, it may result from modifications of cellular proteins during storage at 35°C. Heterogeneous modifications such as lipidation (the covalent binding of a lipid group to a peptide chain), glycosylation and glycation can induce streaking in proteins pattern during bi-dimensional electrophoresis. As explained in the previous section, the development of fluorescent compounds in stored cells is already a sign of glycation.

**Figure 7:**
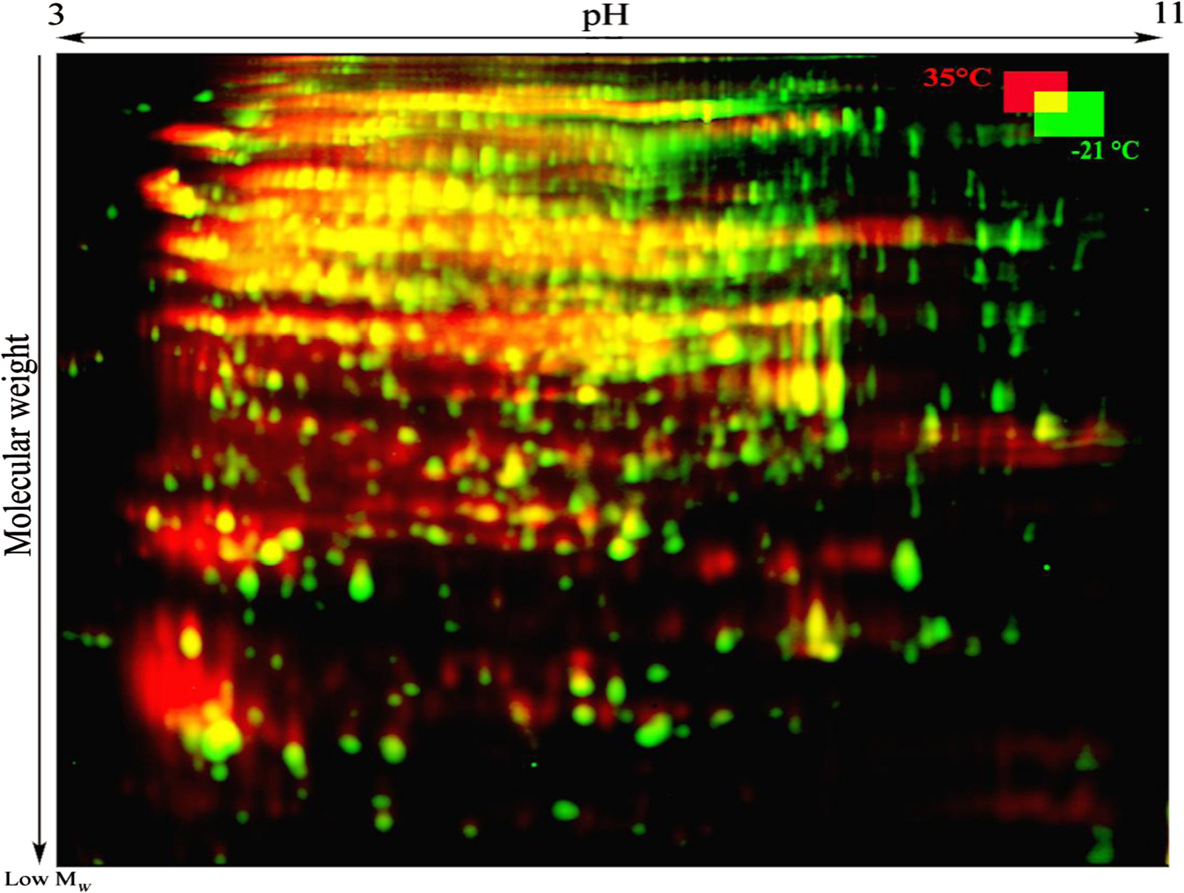
**Comparison between the proteome of freeze-dried *A. senegalensis *preserved at −21 and 35°C for 12 months.** The green and red spots indicate CyDye-labelled proteins from the cells kept at −21 and 35°C, respectively. Trains of red spots are mostly observed in the protein profile of cells stored at 35°C.

Using 2D-DiGE, it was possible to detect changes in molecular weight and shift in Isoelectric point (pI) of cellular proteins. Visual assessment of the 2D protein patterns revealed significant changes that appeared as red or green spots (Figure 7). To quantify changes in protein abundance and position, measurement of all fluorescence-labeled proteins spots was conducted with the Decyder software. Two kinds of modifications were observed during storage at 35°C: a change in the abundance of proteins and a slight or considerable shift toward acidic pI. However, as already mentioned the determination of exact change in abundance of proteins during storage was not feasible due to the extensive formation of spot trains and streaking.

Table 2 lists three proteins with considerable shifts of pI for the cells stored at 35°C. They were found on the acidic side of the gel (pH 4.5-5) although their identification by MALDI-TOF-MS/MS showed that they are ribosomal proteins with theoretical pI between 10.5-11.5. Thus significant modifications occurred causing the displacement of proteins from basic to acidic side of the gel. Modifications that may cause protein pI-shift include protein truncation, acetylation, phosphorylation, or glycosylation (or glycation) [41]. Among these modifications, glycation is more likely to happen since: first the molecular weights did not change excluding a significant truncation of these proteins, second upon phosphorylation, proteins with basic pI to shift only moderately to acidic pI [41], and finally the identification by MALDI-TOF-MS/MS did not reveal any acetylation.

**Table 2:** **Some cellular proteins modifications of freeze-dried *A. senegalensis *** **stored at 35°C for 12 months**

**Master no.**	**Accession no.**	**Proteins**	**Modifications**	**MW (kD)**	**pI**^**1**^	**Modified amino acid and site of carbonylation**	**Score/number of peptides/Percentage of coverage**
***Shift of pI***							
1254	gi|258542052	LSU ribosomal protein L17P	Shift of pI to about 4.5-5	15.5	10.8	---	254/6/36
1456	gi|258542952	LSU ribosomal protein L21P	Shift of’ pI to about 4.5-5	13.1	11.4	---	196/4/31
1697	gi|258542032	LSU ribosomal protein L22P	Shift of’ pI to about 4.5-5	15	11.5	---	229/4/30.9
***Carbonylated proteins***							
1581	gi|258542025	Translation elongation factor Tu	Threonine carbonylation	43.3	5.1	Thr: 111	83/5/50
934	gi|258542743	Translation elongation factor G	Proline carbonylation	76.9	5.2	Pro: 651, 681, 683	85/13/20
914	gi|258542743	Translation elongation factor G	Proline carbonylation	76.9	5.2	Pro: 681 and 683	122/13/24
1313	gi|258541164	SSU ribosomal protein S1	Proline carbonylation	63	5.2	Pro: 262	80/7/15
773	gi|258542981	Pyruvate phosphate dikinase	Proline carbonylation Threonine carbonylation Arginine carbonylation	96.7	5.5	Pro: 758, 623, 670	170/21/17
						Thr: 290 Arg:579	
1552	gi|271502383	Putative lipoprotein	Threonine carbonylation	6.6	9.86	Thr: 9	79/5/96
1368	gi|251798976	PBS lyase	Threonine carbonylation Lysine carbonylation	42.1	6.72	Thr: 373	86/8/24
						Lys: 86, 236	
1392	gi|258541222	ATP synthase F1 beta subunit	Proline carbonylation Arginine carbonylation	52.6	4.6	Pro: 238, 359, 364	125/15/39
						Arg: 68, 289	
1402	gi|258541220	ATP synthase F1 alpha subunit	Proline carbonylation	55.4	5.4	Pro: 5	79/9/20
1540	gi|258542167	Acetyl-CoA hydrolase	Proline carbonylation	54.9	6.2	Pro: 460, 464	88/10/24

^1^The theoretical iso-electric point.

### Carbonylation of proteins increases massively during storage

**Figure 8:**
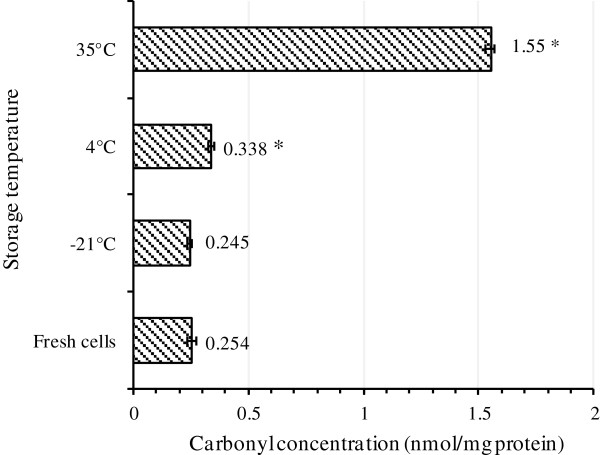
**Quantification of total cellular carbonylated proteins by ELISA test.** The amount of carbonylated proteins increased with storage temperature. For each kind of cell, at least three independent extractions were performed and carbonylated proteins were quantified in triplicates. Error bar represents the standard deviation. The asterisk shows significant difference (*p* < 0.05) between the carbonylated protein content of fresh cells and the carbonylated protein content of the cells kept at different temperature.

Oxidation of side chains of proline, of Proline, Arginine, Lysine and Threonine leads to production of carbonyl groups (aldehyde and ketones) [42]. Since carbonyl groups are chemically stable, they can be used as biomarkers for detection of protein oxidation [42]. In addition, multiple oxidative agents such as ROS or indirect reactions caused by secondary by-products of oxidative stress can lead to production of carbonyl groups [21,43-45], however one of the disadvantage of protein carbonyl as biomarker is that they are nonspecific oxidation markers [46]. It is also noticeable that Methionine (Met) and Cysteine (Cys) are the amino acids most prone to oxidative attacks, and oxidation of other amino acids requires more stringent conditions [14]. Thus, it can be assumed that disulfide bonds and Met sulphoxide residues are formed before or simultaneously with the formation of carbonyl groups in proteins [14]. Total content of carbonylated proteins was quantified by an ELISA method. As Figure 8 shows, the total amount of carbonylated proteins increased dramatically in freeze-dried cells kept at 35°C whereas the cells before freeze-drying (fresh cells) or the cells kept at −21°C did not show any significant difference in the content of carbonylated proteins. For the cells kept at 4°C, the amount of carbonylated proteins was moderately higher than the cells before freeze-drying.

**Figure 9:**
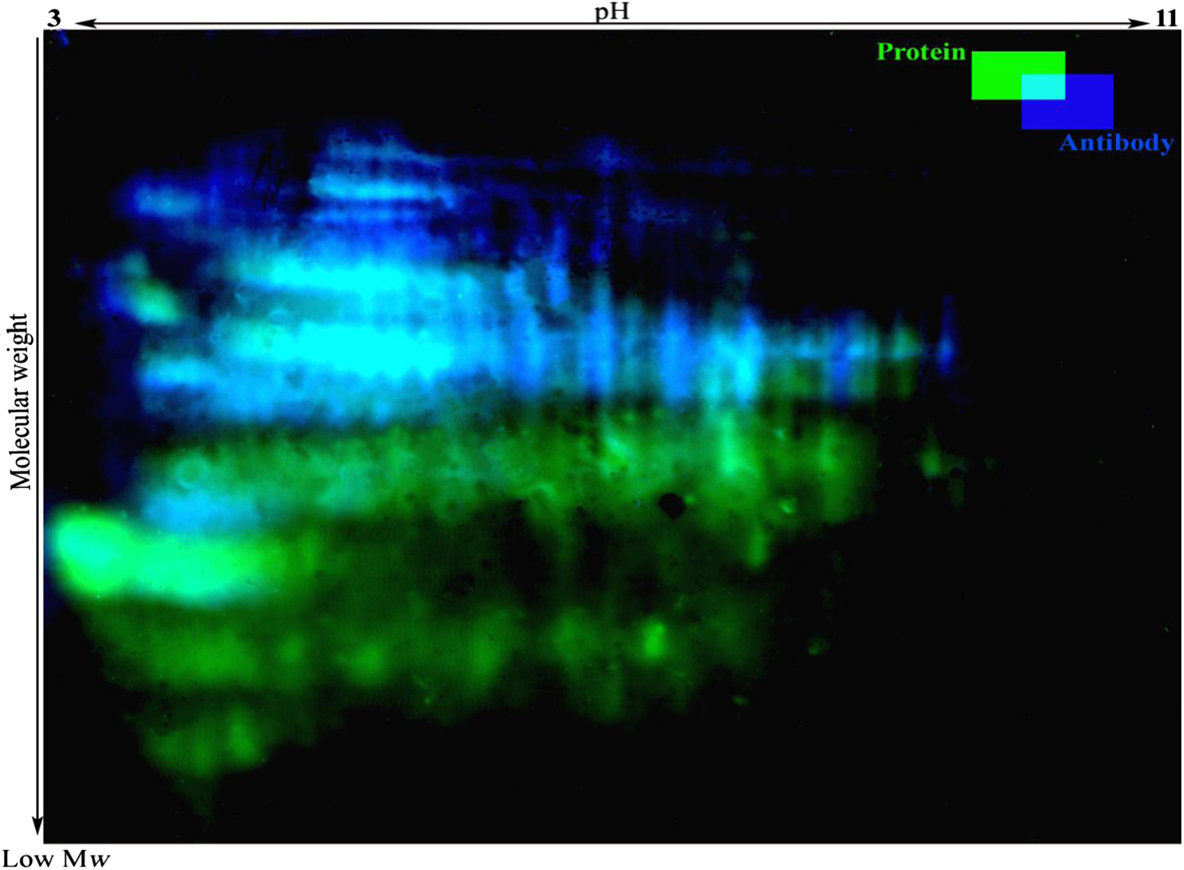
**Protein carbonyl patterns of freeze-dried *A. senegalensis *kept at 35°C for 12 months.** Total proteins were stained with Cy3 (green) and were separated by 2D-DiGE. Following transfer to PVDF membrane, the carbonyl groups in proteins were analyzed by immune-detection using a Cy2-coupled assay (blue).

To determine the effect of storage temperature on the extent of carbonylation of cellular proteins, 2D-DiGE followed by Western blotting was used. Immunoblotting of 2, 4-dinitrophenyl hydrazine (DNPH)-derivatized proteins revealed that various proteins were slightly or heavily carbonylated in cells. Without DNPH derivatization, no spot was detected indicating that the proteins did not react with anti-DNP antibody. In addition, Cy3 and Cy5 dyes used for detection of sample proteins and internal standard proteins respectively, did not emit light when illuminated with 488 nm laser, thus there was no interference between total protein staining and antibody staining in the immunoblot procedure (Figure 9).

**Figure 10:**
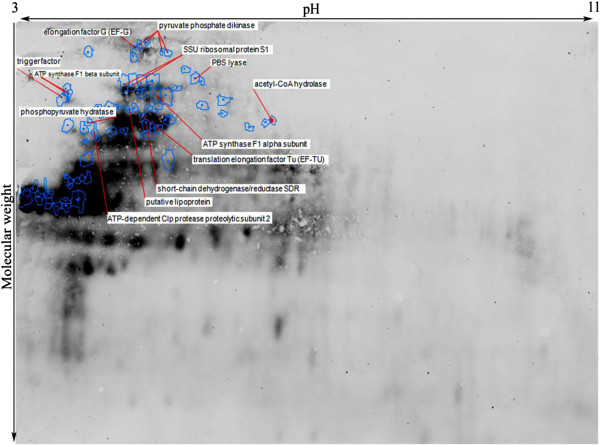
**Identification of carbonylated proteins of freeze-dried *A. senegalensis *stored at 35°C after separation by 2D-electrophoresis.** A preparative gel and a western blot gel containing the same sample were run in parallel. Anti-DNP antibody staining was realized on the WB to localizethe carbonylated proteins spots that were then reported on the preparative gel image, as indicated by the blue spots. Eighty-eight proteins which underwent carbonylation were then picked from the preparative gel and analyzed by mass spectroscopy. Thirteen different proteins were identified as indicated on the gel image, of which 10 are carbonylated proteins with modifications in Arginine, Lysine, Proline and Threonine as listed on Table 2.

As shown in Figures 9 and 10, most of the high molecular weight proteins were carbonylated regardless of their pI whereas carbonylation process did not affect low molecular weight proteins or very basic ones.

By comparing the results of ELISA and western blotting, it was found that the results of Western blotting for samples kept at −21 and 35°C are in accordance with the results of ELISA test. In contrast, a contradiction was observed for cells stored at 4°C. As already explained, a moderate increase in the amount of carbonylated proteins was observed by ELISA method for these cells, however no carbonylated protein were detected using 2D-DiGE followed by western blotting. It is believed that the latter method has significantly more sensitivity and specificity than all other total carbonyl assays such as spectrophotometric DNPH assay and ELISA method [14]. It should however be noted that there is a detection limit for oxidized proteins by immune-blotting methods, and oxidized proteins in low quantity cannot be detected easily [47]. Therefore, it can be assumed that some proteins may undergo very slight carbonylation which is not detectable by these methods. In addition, as already discussed, some other protein oxidation reactions may occur prior to carbonylation which may cause VBNC formation in cells. In order to identify the proteins that were oxidized when cells were kept at 35°C, 88 protein spots were selected and picked from preparative gel maps that had been matched to the 2-4-DNP-derivatized protein spots maps and subjected to identification by MSMS analysis. Proteins were identified in only 13 of the 88 selected spots from Figure 10 of which 10 contained carbonylated amino acids (Table 2). This low number technically results from the limited coverage of sequence (generally between 15 and 50%) that was achieved allowing only a small number of peptides to be analyzed. Thus, the lack of detection of carbonylated residues by MSMS analysis does not necessarily means that the identified proteins were not carbonylated. Lysine, Arginine, Threonine and Proline were identified as target amino acids where carbonylation occurred. Both structural proteins and enzymes were found among the carbonylated proteins.

Our results are considerably consistent with the studies showing the susceptibility of cell envelope protein and translational proteins to oxidative stress [47-50]. It has already been shown that exposure of *E. coli* to different stress conditions such as H_2_O_2_, iron overloading or super oxide generating compounds causes carbonylation of Elongation factor G (EF-G), outer membrane protein A (ompA), β-subunit of F0F1- ATPase, heat shock proteins and enolase. In addition, following such oxidative stress, the viability was affected to different extents depending on the type of exerted stress [47]. One group of carbonylated proteins identified in this study indeed belongs to the cell translation machine (Figure 10 and Table 2). SSU ribosomal protein S1 presumed function is to participate in the initiation of translation, possibly by binding mRNA and directing it to the ribosome [51]. There are also evidences that heat stress causes important irreversible reactions in the cell ribosomes; however more experimental evidence is still needed to determine whether or not ribosome is the critical component responsible for the thermal death of microorganisms [11,52,53]. Thus, the obtained results can be used as an indication and also confirmation that during cell death at high storage temperature, ribosomal proteins are oxidized.

Other proteins involved in translation process which were also carbonylated during storage of cells at 35°C are Elongation factor G (EF-G) and Elongation factor TU (EF-TU). In the protein synthesis process by ribosome, loading of tRNA into the A site of 50S subunit is assisted by the elongation factor EF-Tu. Elongation factor G (EF-G) is involved in translocation of the tRNA holding the polypeptide [54]. Recently, it has been shown that the latter protein is very susceptible to oxidation during oxidative stress in *E. coli*. Under in vitro conditions, treatment of EF-G with H_2_O_2_ resulted in a complete loss of activity due to oxidation of cysteine residues [55]. In our previous study on storage of freeze-dried *A. senegalensis*, we have shown that cell envelope integrity was subjected to changes during freeze-drying process and storage at 35°C [4]. Those findings are consistent with the observations of the present study and some other studies stating that essential targets on which survival depends during ROS stress include membrane lipid integrity and ROS-susceptible proteins, including proteins required for faithful translation of mRNA [56].

Another group of oxidized proteins identified in this study is involved in cell energy generation. The α and β subunits of F1 ATP synthase were detected as heavily carbonylated proteins (Table 2). ATPases consist of two components, a multiprotein cytoplasmic complex called F1 that carries out the chemical function (ATP synthesis), connected to a membrane-integrated component called F0 that carries out the ion-translocating function [54]. As already mentioned, the respiration system of cells kept at 4 and 35°C was affected during storage (Figure 1). In addition, in our previous study on stored freeze-dried *A. senegalensis,* we observed that cellular respiration was impaired during storage at high temperature [4], thus the results of the present study suggest that respiration disability of these cells may be due to defective ATP synthase involved in respiration chain.

In our previous study on storage of freeze-dried *A. senegalensis*, we have also shown that the cell envelope integrity was subjected to changes during freeze-drying process and storage at 35°C [4]. Those findings are consistent with the observations of the present study and some other studies stating essential targets on which survival depends during ROS stress include membrane lipid integrity and ROS-susceptible proteins, including proteins required for faithful translation of mRNA [56]. According to the obtained results from the ELISA test and immunoblotting, with regard to the results of viability and culturability (Figure 2), it can be inferred that carbonylation of proteins is not the main reason for the entrance of bacteria to VBNC at low storage temperature. Thus, it can be assumed that other deleterious reactions preceding carbonylation can lead to the death or VBNC formation at low temperatures.

### Solubility of cellular proteins is reduced during storage

We found that solubility of cellular proteins decreases at high storage temperature (35°C). Total cellular proteins of stored cells were extracted using different solutions (low salt (LS), High Salt (HS), Ethanol, NaOH) and the amount of proteins in each fraction was determined by the Bradford method. As shown on Table 3, the amount of released proteins (RP) from cells kept at various temperatures after washing in phosphate buffer (KPB) did not show significant differences (*p* > 0.05, range 0.99), indicating that the discharge of proteins from damaged cells did not occur after storage. However, the amount of total soluble proteins which remained in the supernatant after ultra-sonication in low salt fraction (LS) decreased as the storage temperature increased. In other words, higher storage temperature caused protein insolubility. Storage of cells at lower temperature (−20, +4°C) for long time (12 months) did not cause protein insolubility in LS and NaOH fraction (*p* > 0.05, range 0.62) whereas storage at 35°C resulted in lower concentration of soluble proteins in LS and NaOH solutions (*p* < 0.05, range 0.004). A comparison between the cells kept at −21°C and 35°C shows that preservation of cells at 35°C caused about 21.3% insoluble proteins in LS fraction. As the moisture content of freeze-dried cells was constant during storage, it seems that the change in protein solubility was mainly related to the storage temperature.

**Table 3:** Soluble protein contents (mg/g dried cells) of cells kept at different storage temperatures for 12 months

**Fractions**	**Storage temperature**		
	**−21°C**	**+4°C**	**+35°C**
**KPB**	9.54 ± 1.41	11.05 ± 3.33	11.14 ± 2.98
**LS**	74.1 ± 6.15	66.67 ± 4.05	58.75 ± 6.38
**HS**	0.74 ± 0.43	1.26 ± 0.21	1.25 ±0.18
**Ethanol**	0.44 ± 0.18	0.39 ± 0.13	0.24 ± 0.12
**NaOH**	42.49 ± 0.71	41.99 ± 3.29	34.99 ± 5.65

KPB: 50 mM potassium phosphate buffer solution, pH 6.8; LS: low salt buffer; HS: high salt buffer. Ethanol 70% (v/v) and NaOH 0.1 M. Data are presented as mean ± SD.

One direct consequence of oxidative damage (such as carbonylation) to proteins is a change in protein solubility. High molecular-weight aggregates are formed when proteins are heavily carbonylated whereas the proteasomal system only moderately degrades carbonylated proteins [57]. Since carbonylation of proteins is an irreversible and irreparable oxidative damage [57,58], the generated aggregates accumulate as damaged or unfolded proteins [57]. An important question is whether protein insolubility which is observed in many aging processes (as well as in the present study) is also responsible for the death of bacterial cells into VBNC state. Whatever the causal relationship between protein oxidation and temperature, oxidative stress has been recognized to play a major role in cell viability by direct or indirect protein modifications [44]. Maisonneuve and coworkers have shown the presence of aggregated proteins in aerobically growing healthy *E. coli.* As the level of aggregation was correlated with the amount of ROS produced during growth, they finally concluded that aggregates may function as temporary trash organelles for detoxifications [59]. In addition, it has been shown that protein aggregates increase during population senescence of *E. coli*, and reach a maximum in stationary phase. The amount of protein aggregates is proportional to the ratio of dead cells [60]. Cao and et al. showed that viability of invading bacteria to plant cells was reduced through oxidation of special membrane transporters [61]. It was also shown that *E. coli* VBNC state during stationary phase is preceded by damage to proteins such as carbonylation of proteins which affects various bacterial compartments and proteins [62].

According to these results and in general agreements with earlier studies, it can be assumed that insolubility of proteins which can be one of the consequence of protein carbonylation increases during senescence of bacterial population in storage period, and thus can be considered as a cause of death.

### Changes in fatty acid profile may enhance protein modification

As already discussed, the proteome of cells kept at 35°C showed trains of spots or streaking (Figures 5 and 6).

**Figure 11:**
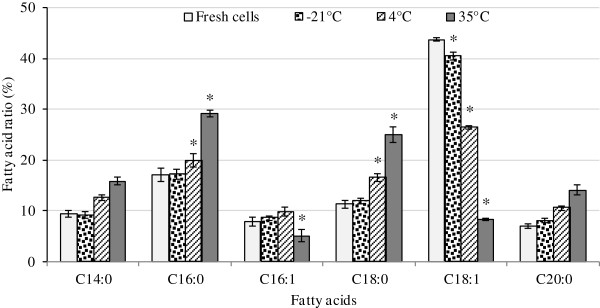
**Fatty acid content of freeze-dried *A. senegalensis *cells stored at different temperatures for 12 months.** The ratio of unsaturated fatty acid to total extracted fatty acid decreased during storage at high temperature. Error bar shows the standard deviation. Asterisk shows the significant difference (*p < 0.05*) between the content of fatty acid for fresh cells and stored cells.

In the present study, the fatty acid content of fresh and freeze-dried cells was analyzed using gas chromatography method. As Figure 11 shows, Palmitoleic acid (C16:1) and Oleic acid (C18:1n9c) were detected as the main unsaturated fatty acids in fresh cells before drying. Oleic acid (C18:1n9c) was the predominant fatty acid identified by this method. After storage of the cells at different temperatures, the fatty acid profile was subjected to changes, and the unsaturated fatty acid content of cells kept at 35°C decreased considerably (*p* < 0.05, range 0.002). In contrast, the percentage of some saturated fatty acids increased significantly (*p* < 0.05, range 0.001) in the cells kept at 35°C.

The same trend of fatty acid change was observed for cells kept at lower storage temperature; but, the extent of change was temperature dependent. The percentage of oleic acid moderately decreased during storage at 4°C (*p* < 0.05) and was even less reduced at −21°C (*p* < 0.05, range 0.004). Change of fatty acid ratio (unsaturated/saturated) of different lipid products during storage conditions have been reported by many authors [11,30,63-65]. It is well-known that the loss of bacterial viability during storage is increased following lipid oxidation [11,49]. α, β-unsaturated alkenals such as 4-hydroxynonenal produced during the peroxidation of polyunsaturated fatty acids have been shown to react with proteins [22] and to form stable covalent thiolether adducts carrying a carbonyl function [44].

Considering the storage condition of our samples (normal atmosphere, low and high temperature) and the intrinsic factors in the formulation (presence of glucose, and spent growth culture media), several sources of ROS formation might exist. Formation of ROS during freeze-drying process and storage of freeze-dried products has indeed been reported [34,35]. At low moisture content, non-enzymatic reactions are known to occur, such as Amadori and Maillard reactions [66], as well as lipid peroxidation [34,66]. Enzymatic oxidation of lipids through lipoxygenase is also possible in low water activity (about 0.4) [66]. Indeed, it is widely accepted that presence of lipid moiety, specially unsaturated lipids may enhance free radical-mediated oxidative damage [34,67] it has also been shown that presence of lipids in freeze-drying formulation can enhance the ROS formation during storage [34].

## Conclusions

This work demonstrated that the protein content of freeze-dried *A. senegalensis* is subjected to different changes during storage period. In addition, storage temperature affects the total cellular proteome significantly. Although many studies attempted to improve viability in bacterial starter, modifications of bacterial cellular proteins during storage of freeze-dried starter have not been considered in details. An association between storage temperature and protein carbonylation was established in our study. Moreover, we showed that samples mainly composed of dead cells contained higher amount of carbonylated proteins including some critical proteins such as ribosomal proteins or proteins involved in energy generation. Moreover, since protein carbonylation is a reaction which normally occur after other oxidative reactions, it seems that some other reactions (such as lipid peroxidation or protein lipidation and glycation) happen prior or in parallel to carbonylation. These reactions may be involved in VBNC formation even at very low storage temperature.

Formation of fluorescent substances in the stored cells, the changed pattern of 2D-DiGE and the modifications in composition of cellular fatty acids can be considered as indications of Maillard reaction progression, AGEs formation and protein lipidation. Therefore, from a practical point of view, it is first suggested to decrease the residues of spent growth medium and the metals content in the freeze-drying formula (e.g. by washing cells with appropriate buffers before freezing process to eliminate the fermentation supernatant). Secondly, because a part of the cells already has been entered into VBNC state during drying process, it seems that the deterioration reactions were first initiated during freeze-drying process and enhanced during storage. Accordingly, to diminish the deterioration reactions during storage period, any process that may causes bacterial injuries during fermentation and freeze-drying process must be avoided. Finally, since the deterioration reactions are considerably temperature dependent it is recommended to keep the freeze-dried bacteria at low temperature.

## Materials and methods

### Microorganisms and production of biomass

*A. senegalensis* CWBI-B418T (=LMG 23690 T = DSM 18889 T), a thermo-tolerant acetic acid bacterium, isolated from mango fruit in Walloon Center of Industrial biology (CWBI), was used through this study [8].

*A. senegalensis* was grown in a 15 L laboratory-scale bioreactor (Bio Biolaffite, France) containing glucose (20 g/l), yeast extract (7.5 g/l), MgSO_4_.7 H_2_O (1 g/l), and (NH4)_2_HPO_4_ (1 g/l), K_2_HPO_4_ (1 g/l). The fermentation was performed under conditions previously described [4].

### Production and storage of freeze-dried cells

Production of freeze-dried starter culture was performed as previously described [4] except that the cells were not mixed with mannitol. Instead, the cells were mixed with culture supernatant to reach the right dry weight. Freeze-dried cells were crushed and then dispensed into glass vials. The vials were sealed with septum and caps. They were then kept at three different temperature (−21, 4 and 35°C) up to 12 months in the dark.

### Determination of total cell number and culturable cells after freeze-drying process

Total cell number of fresh cells and rehydrated cells was determined by using Brucker slides (Lo-Laboroptic Ltd, Lancing, UK). Briefly, 100 mg of freeze-dried cells were resuspended in 10 ml of GY medium. After 5 min of incubation at 30°C, it was mixed vigorously. The number of cells was then counted by using phase contrast microscope (Olympus, Tokyo, Japan).

Culturable cells in freeze-dried samples were determined by using spread plate technique. GY medium was used as the medium for enumeration of culturable cells. GY agar medium contained: glucose 20 g/l, yeast extract 7.5 g/l, MgSO_4_.7 H_2_O 1 g/l, and (NH4)_2_HPO_4_ 1 g/l, KH_2_PO_4_ 1 g/l, agar 15 g/l. pH of medium was set at 6.0 ± 0.05.

GY broth (the same components as GY agar) were used as rehydration medium and diluting medium as well.

### Determination of total dehydrogenase activity of stored cells

5-cyano-2,3-ditolyl tetrazolium chloride (CTC) (λ_ex_ 450 nm; λ_em_ 630 nm) and thiazole orange (TO) (λ_***ex***_ 509 nm; λ_*em*_ 530 nm**)** were used to determine the total dehydrogenase activity. CTC is a redox dye which is converted to a solid formazan dye by cellular respiratory chain. Solid formazan emits red fluorescence.

A stock solution of 50 mM CTC in filtrated deionized water, and a stock solution of 42 μmol/L TO in DMSO were prepared. The TO solution was diluted 10 times in DMSO just before staining of cells [4,5]. All experiments were performed using a BD FACSCalibur™ flow cytometer (Becton, Dickinson, USA) equipped with an air-cooled argon laser and a red diode laser. Data were analyzed using BD CellQuest™ Pro software (Becton, Dickinson, USA). Instrument settings and staining procedure were performed as already described [4]. Briefly, freeze-dried *A. senegalensis* were rehydrated and harvested by centrifugation, and the pellets were washed twice with saline phosphate buffer solution containing 18 mM glucose (PBSG). 45 μl of CTC solution was mixed with 450 μl of cell suspension and incubated at 30°C for 90 min on shaker (130 rpm) in the dark, then 5 μl of diluted TO was added to each sample and incubation was continued for 5 min at 30°C in the dark before passing the samples to FACS Cytometry.

### Assessment of fluorescent substance formation in stored cells

Auto-fluorescence of cells was detected according to the procedure explained by Sheehy [68]. Briefly, freeze-dried cells were suspended in PBS (100 mM, pH 7.4) for 10 min and washed three times to remove extracellular components. Then, the cell suspensions were delivered to BD FACSCalibur™ flow cytometer (Becton, Dickinson, USA) at low flow rate, corresponding to 500–700 events/s (in the Forward Scatter vs. Side Scatter plot). The autofluorescence signal was collected on FL3. For each cell population, three independent samples were prepared and introduced to the FACS. The total counted events for each sample were set on 55000.

### Sequential extraction of cellular proteins

30 mg of freeze-dried cells were suspended in 10 ml of 50 mM phosphate buffer (pH 6.8), mixed vigorously and incubated at 25°C for 10 min, and then the cells were washed with the phosphate buffer solution twice. Washed cells were re-suspended in 1 ml of 50 mM Tris–HCl, 200 mM NaCl buffer (pH 8.3) and sonicated (50% power, 5 cycles) for 135 seconds on ice. Afterwards, the sequential extraction was performed in protein free Eppendorf® tubes as follows: 50 mM Tris–HCl, 200 mM NaCl buffer (pH 8.3) (Low Salt, LS) for 20 min on ice (twice); 50 mM Tris– HCl, 1 M NaCl buffer (pH 8.3) (High Salt, HS) for 20 min on ice; 70% (v/v) ethanol for 30 min at 65°C (E); and 0.1 M NaOH solution for 30 min on ice. At each extraction stage, 0.6 μl of protease inhibitor cocktail for general use (Sigma-Aldrich) was added to the solutions. Supernatants were obtained after 10 min centrifugation (14,000 g and 4°C), and then the amount of soluble protein in each fraction was determined by Bradford method.

### 2D-DiGE analysis of freeze-dried cells stored at different temperature

2D-DIGE proteomic analysis has been performed on freeze-dried cells kept at different temperatures for 12 months according to the procedures already used and published by Wislet-Gendebien et al. [69]. Proteins were extracted in a lysis buffer containing 7 M urea (GE Healthcare, Diegem, Belgium), 2 M thio-urea (GE Healthcare), 30 mM Tris (pH 8.5) (GE Healthcare) and 2% ASB14 (Sigma-Aldrich). The supernatant containing the extracted solubilized proteins was precipitated (2-D Clean- Up Kit; GE Healthcare) and proteins resolubilized in lysis buffer were quantified using RC-DC Protein Assay (Bio-Rad). Each 25 μg of sample proteins was labeled with 200 pmol CyDye (GE Healthcare), either Cy3 or Cy5, and left for 30 min in the dark. The labeling reaction was stopped by adding 10 mM lysine for 10 min at 4°C. An internal standard was prepared by mixing equal quantities of all the experimental samples and was labeled with Cy2. Then, all the samples within the experiment were mixed in pairs together with 25 μg of the labeled internal standard and were separated by isoelectric focusing using pH 3–11 (24 cm) IPG strips in an Ettan IPGphor focusing system (GE Healthcare) in the first dimension.

Before initiating the second dimension step, proteins in IPG strips were reduced for 15 min in an equilibration buffer (50 mM Tris–HCl (pH 8.8), 6 M urea, 30% glycerol, 2% SDS) containing 1% DTT and then they were alkylated in the same equilibration buffer containing 5% iodoacetamide. IPG strips were placed on top of classical 12.5% SDS-PAGE gels and the electrophoretic migration was completed in an Ettan Dalt apparatus (GE Healthcare) at 2 W/gel for 30 min, and then 25 W for 18 h. After scanning the gels with a Typhoon 9400 Laser Scanner (GE Healthcare) at three different wavelengths corresponding to the different CyDyes, 2-D gel analysis software (DeCyder version 7.0; GE Healthcare) was used for spot detection, spot quantification relative to the corresponding spot in the internal standard and gel matching. Protein spots that showed a significant variation in their abundance of at least twofold (Student’s t test, p < 0.05) between storage conditions were selected for further identification and were automatically picked from preparative gels run in parallel with the Ettan Spot Picker (GE Healthcare). Proteins in spots were identified by MALDI-TOF-MS-MS at the GIGA-Research Proteomic platform. Gels plugs were subjected to automatic tryptic digestion (PROTEINEER dp automated digester; Bruker Daltonics, Bremen, Germany). Gel pieces were washed three times in 50 mM NH_4_HCO_3_ followed by 50% ACN/50 mM NH_4_HCO_3_. Two other washes were carried out with 100% ACN to dehydrate the gels. In-gel digestion was performed with freshly activated trypsin (Roche, Basel, Switzerland) at a concentration of 10 ng l^-1^ in 50 mM NH_4_HCO_3_/5% ACN. After rehydration of the gel pieces at 8°C for 60 min, tryptic digestion was carried out at 30°C for 4 h. The resulting digested peptides were extracted with 1% trifluoroacetic acid (TFA) for 30 min at 20°C with occasional shaking. A volume of 3 μl of protein digests was adsorbed for 3 min on Prespotted AnchorChip plates with a-Cyano- 4-hydroxycinnamic acid (CHCA) as a matrix, using the PROTEINEER dp automat. Then the spots were briefly desalted with 10 mM dihydrogen ammonium phosphate in 0.1% TFA. MS fingerprints of the samples were acquired using the Ultraflex II MALDI-TOF–TOF mass spectrometer (Bruker Daltonics) in the mass range 700–3,500 Da. The Peptide Mass Fingerprinting (PMFs) was searched against the NCBI database. The variable and fixed modifications were methionine oxidation and cysteine carbamylation, respectively, with a maximum number of missed cleavages of 1. Mass precision tolerance error was set to 100 ppm. Peaks with the highest intensities, obtained in TOF/MS mode, were next analyzed by LIFT MS/MS. Proteins were identified with the Biotools 3.0 software (Bruker Daltonics) using the Mascot search engine (Matrix Science, Boston, MA, USA).

### Quantification of carbonylated proteins

Carbonyl assay was performed according to the procedure explained in the manual of protein carbonyl ELISA kit (Enzo® Life science catalog # ALX-850-312-KI01). Briefly, after extraction of total cellular proteins in extraction buffer (Tris 100 mM pH 7.4, 100 mM NaCl, 1 mM EDTA, 1% Triton X100, 0.5% Sodium Deoxycholate) by sonicator (50% power, 6 cycles, 5 min, on ice) and removal of DNA by streptomycin solution, the extracted proteins were reacted with Dinitrophenyl hydrazine (DNP); then the proteins were nonspecifically adsorbed to an ELISA plate. Unconjugated DNP and non-protein constituents were washed away. The adsorbed proteins were probed with biotinylated anti-DNP antibody followed by streptavidin-linked horseradish peroxidase. Adsorbances were related to a standard curve prepared with serum albumin containing increasing proportions of hypochlorous acid-oxidized proteins that has been calibrated colorimetrically.

### Determination of carbonylated proteins by western blotting

For detection and identification of carbonylated proteins in freeze-dried cells, a modified 2D-DiGE analysis (200 μg of one individual sample per gel together with 25 μg internal standard) was run, followed by western blotting. 2D-DiGE procedure was performed as mentioned in the previous section except that before running the second dimension, the IPG strips were incubated in a 10 mM 2,4-dinitrophenyl hydrazine (DNPH) solution for 20 min at 25°C. The IPG strips were then rinsed with Tris base/glycerol (2 M/30% (v/v)) solution and equilibration buffer. Each strip was then treated for reduction/alkylation and the second dimension of electrophoresis was run as mentioned above.

Three gels prepared from each cell samples (nine gels for the three storage temperature) were transferred to activated PVDF membranes. Carbonyl groups in the transferred derivatized proteins were immunodetected with anti-2,4-DNP primary antibody (1/500) overnight at 4°C in skimmed milk-TTBs buffer. Then, the membranes were washed three times in TTBS buffer and incubated with the second antibody (1/2500) at room temperature for 1 h. Finally, after three washings with TBS buffer, the membranes were scanned with a Typhoon 9400 Laser Scanner (GE Healthcare) at three different wavelengths corresponding to the different CyDyes and Anti-DNP antibody. 2-D gel analysis software (DeCyder version 7.0; GE Healthcare) was used for spot detection, gel matching and spot quantification relative to the corresponding spot in the internal standard.

### Total fatty acid analysis

Analysis of the fatty acid content was performed after trans-esterification of extracted fatty acid. Briefly, 100 mg of freeze-dried cells was suspended in 10 ml of 50 mM phosphate buffer (pH 6.8), mixed vigorously and incubated at 25°C for 10 min, and then the cells were washed twice with the same phosphate buffer solution. The washed cells were then mixed with 300 μl of a solution containing Chloroform/Methanol (2:1). After addition of glass beads to the cell suspension, the cells underwent three cycle of heat shock (Liquid nitrogen-37°C). They were then mixed vigorously for 10 min. The supernatant was mixed with 1 ml of methylation mixture (33% methanolic 3 N HCl, 67% methanol, 10 μg/ml BTH), and methylation was performed at 85°C for 35 min. After cooling of the samples, 900 μl of 0.9% NaCl solution was added to each sample, and finally the lipid phase was dissolved in Heptan.

The gas chromatography analysis of the fatty acids contents was performed with a GC-2010 Shimadzu Gas chromatograph equipped with a SGE-capillary BPX70 column (30 m length). Supelco-FAME mix-37 (Sigma–Aldrich) was used as standard for fatty acids identifications and quantifications.

### Statistical analysis

All the experiments were performed at least in three independent replicates. Kolmogorov-Smirnov normality test was used for checking the normality of data. The test showed that in all the experiments, the data are approximately normally distributed. Two-Factors Repeated Measures ANOVA was performed on the data by R.3.0.10 Software. After doing ANOVA, we performed *Duncan’s* multiple range *test* (MRT) to investigate significant levels for the difference between any pair of means, regardless of whether a significant F resulted from ANOVA.

## Competing interests

The authors declare that they have no competing interests.

## Authors’ contributions

RS designed the experimental setup and carried out the fermentation, freeze-drying process, flow cytometry and proteomic analyses. RS also prepared the manuscript, figures and tables. RZ contributed in fermentation, ELISA analysis and helped in the revision of the manuscript. AB developed the protocol for Immunoblotting techniques. MB participated in the statistical analysis and interpretation of the data. PL supervised the proteomic analysis, and revised the manuscript. PT and FD supervised the whole work and revised the manuscript. All authors read and approved the final manuscript.

## References

[B1] Holzapfel WH Appropriate starter culture technologies for small-scale fermentation in developing countries Int J Food Microbiol 2002 75 197 212 10.1016/S0168-1605(01)00707-3 12036143

[B2] Sokollek SJ Hammes WP Description of a starter culture preparation for vinegar fermentation Syst Appl Microbiol 1997 20 481 491 10.1016/S0723-2020(97)80017-3

[B3] Azuma Y Hosoyama A Matsutani M Furuya N Horikawa H Harada T Hirakawa H Kuhara S Matsushita K Fujita N Shirai M Whole-genome analyses reveal genetic instability of *Acetobacter pasteurianus* Nucleic Acids Res 2009 37 5768 5783 10.1093/nar/gkp612 19638423 PMC2761278

[B4] Shafiei R Delvigne F Thonart P Flow-cytometric assessment of damages to *Acetobacter senegalensis* during freeze-drying process and storage Acetic Acid Bacteria 2013 2 s1 e10

[B5] Shafiei R Delvigne F Babanezhad M Thonart P Evaluation of viability and growth of *Acetobacter senegalensis* under different stress conditions Int J Food Microbiol 2013 163 204 213 10.1016/j.ijfoodmicro.2013.03.011 23562697

[B6] Gullo M Mamlouk D De Vero L Giudici P *Acetobacter pasteurianus* strain AB0220: cultivability and phenotypic stability over 9 years of preservation Curr Microbiol 2012 64 576 580 10.1007/s00284-012-0112-9 22441885

[B7] Ndoye B Weekers F Diawara B Guiro AT Thonart P Survival and preservation after freeze-drying process of thermoresistant acetic acid bacteria isolated from tropical products of Subsaharan Africa J Food Eng 2007 79 1374 1382 10.1016/j.jfoodeng.2006.04.036

[B8] Ndoye B Lebecque S Dubois-Dauphin R Tounkara L Guiro AT Kere C Diawara B Thonart P Thermoresistant properties of acetic acids bacteria isolated from tropical products of Sub-Saharan Africa and destined to industrial vinegar Enzyme Microb Technol 2006 39 916 923 10.1016/j.enzmictec.2006.01.020

[B9] Santivarangkna C Kulozik U Foerst P Alternative drying processes for the industrial preservation of lactic acid starter cultures Biotechnol Prog 2007 23 302 315 10.1021/bp060268f 17305363

[B10] Miyamoto-Shinohara Y Imaizumi T Sukenobe J Murakami Y Kawamura S Komatsu Y Survival rate of microbes after freeze-drying and long-term storage Cryobiology 2000 41 251 255 10.1006/cryo.2000.2282 11161557

[B11] Santivarangkna C Kulozik U Foerst P Inactivation mechanisms of lactic acid starter cultures preserved by drying processes J Appl Microbiol 2008 105 1 13 10.1111/j.1365-2672.2008.03744.x 18266696

[B12] Morgan CA Herman N White PA Vesey G Preservation of micro-organisms by drying: a review J Microbiol Methods 2006 66 183 193 10.1016/j.mimet.2006.02.017 16632005

[B13] Lievense LC Van’t Riet K Convective drying of bacteria Adv Biochem Eng Biotechnol 1994 51 72 86 8165952

[B14] Shacter E Quantification and significance of protein oxidation in biological samples Drug Metab Rev 2000 32 307 326 10.1081/DMR-100102336 11139131

[B15] Lund MN Heinonen M Baron CP Estévez M Protein oxidation in muscle foods: a review Mol Nutr Food Res 2011 55 83 95 10.1002/mnfr.201000453 21207515

[B16] Ragoonanan V Aksan A Protein Stabilization Transfus Med Hemother 2007 34 246 252 10.1159/000104678

[B17] Fredrickson JK Li SMW Gaidamakova EK Matrosova VY Zhai M Sulloway HM Scholten JC Brown MG Balkwill DL Daly MJ Protein oxidation: key to bacterial desiccation resistance? ISME J 2008 2 393 403 10.1038/ismej.2007.116 18273068

[B18] Castellión M Matiacevich S Buera P Maldonado S Protein deterioration and longevity of quinoa seeds during long-term storage Food Chem 2010 121 952 958 10.1016/j.foodchem.2010.01.025

[B19] Claudette Job LR Lovigny Y Belghazi M Job D Patterns of protein oxidation in Arabidopsis seeds and during germination Plant Physiol 2005 138 790 802 10.1104/pp.105.062778 15908592 PMC1150397

[B20] Linares MA Marín-García P Méndez D Puyet A Diez A Bautista JM Proteomic approaches to identifying carbonylated proteins in brain tissue J Proteome Res 2011 10 1719 1727 10.1021/pr101014e 21235272

[B21] Dalle-Donne I Giustarini D Colombo R Rossi R Milzani A Protein carbonylation in human diseases Trends Mol Med 2003 9 169 176 10.1016/S1471-4914(03)00031-5 12727143

[B22] Møller IM Jensen PE Hansson A Oxidative modifications to cellular components in plants Annu Rev Plant Biol 2007 58 459 481 10.1146/annurev.arplant.58.032806.103946 17288534

[B23] Nystrom T Role of oxidative carbonylation in protein quality control and senescence EMBO J 2005 24 1311 1317 10.1038/sj.emboj.7600599 15775985 PMC1142534

[B24] Jung T Höhn A Grune T Armstrong D Lipofuscin: Detection and quantification by microscopic techniques Advanced Protocols in Oxidative Stress II 2010 594 Humana Press, Springer New York Dordrecht Heidelberg London 173 193 Methods in Molecular Biology. ISBN 978-1-60761-410-4 10.1007/978-1-60761-411-1_13 20072918

[B25] Baynes JW The role of AGEs in aging: causation or correlation Exp Gerontol 2001 36 1527 1537 10.1016/S0531-5565(01)00138-3 11525875

[B26] Murthy UMN Liang Y Kumar PP Sun WQ Non-enzymatic protein modification by the Maillard reaction reduces the activities of scavenging enzymes in *Vigna radiata* Physiol Plant 2002 115 213 220 10.1034/j.1399-3054.2002.1150206.x 12060238

[B27] Murthy UMN Kumar PP Sun WQ Mechanisms of seed ageing under different storage conditions for *Vigna radiata* (L.) Wilczek: lipid peroxidation, sugar hydrolysis, Maillard reactions and their relationship to glass state transition J Exp Bot 2003 54 1057 1067 10.1093/jxb/erg092 12598575

[B28] Kurtmann L Skibsted LH Carlsen CU Browning of freeze-dried probiotic bacteria cultures in relation to loss of viability during storage J Agric Food Chem 2009 57 6736 6741 10.1021/jf901044u 19591471

[B29] Nocker A Fernández PS Montijn R Schuren F Effect of air drying on bacterial viability: a multiparameter viability assessment J Microbiol Methods 2012 90 86 95 10.1016/j.mimet.2012.04.015 22575714

[B30] Coulibaly I Amenan AY Lognay G Fauconnier ML Thonart P Survival of freeze-dried *Leuconostoc mesenteroides* and *Lactobacillus plantarum* related to their cellular fatty acids composition during storage Appl Biochem Biotechnol 2009 157 70 84 10.1007/s12010-008-8240-1 18491235

[B31] Wesche AM Gurtler JB Marks BP Ryser ET Stress, sublethal injury, resuscitation, and virulence of bacterial foodborne pathogens J Food Prot 2009 72 1121 1138 19517746 10.4315/0362-028x-72.5.1121

[B32] Hoefman S Van Hoorde K Boon N Vandamme P De Vos P Heylen K Survival or revival: long-term preservation induces a reversible viable but non-culturable state in methane-oxidizing bacteria PloS One 2012 7 e34196 10.1371/journal.pone.0034196 22539945 PMC3335116

[B33] Vriezen JA de Bruijn FJ Nusslein KR Desiccation induces viable but non-culturable cells in *Sinorhizobium meliloti* 1021 AMB Express 2012 2 6 10.1186/2191-0855-2-6 22260437 PMC3293009

[B34] Molina MD Anchordoquy TJ Degradation of lyophilized lipid/DNA complexes during storage: the role of lipid and reactive oxygen species Biochim Biophys Acta 2008 1778 2119 2126 10.1016/j.bbamem.2008.04.003 18445474

[B35] Molina MC Anchordoquy TJ Metal contaminants promote degradation of lipid/DNA complexes during lyophilization Biochim Biophys Acta Biomembr 2007 1768 669 677 10.1016/j.bbamem.2006.12.004 PMC1851895 17224131

[B36] Narayana Murthy UM Sun WQ Protein modification by Amadori and Maillard reactions during seed storage: roles of sugar hydrolysis and lipid peroxidation J Exp Bot 2000 51 1221 1228 10.1093/jexbot/51.348.1221 10937697

[B37] Cohen-Or I Katz C Ron EZ AGEs secreted by bacteria are involved in the inflammatory response PloS One 2011 6 e17974 10.1371/journal.pone.0017974 21445354 PMC3062560

[B38] Markowicz Bastos D Monaro E Siguemoto E Séfora M Valdez B Maillard reaction products in processed food: Pros and Cons Food Industrial Processes - Methods and Equipment 2012

[B39] Kurtmann L Carlsen CU Skibsted LH Risbo J Water activity-temperature state diagrams of freeze-dried *Lactobacillus acidophilus* (La-5): influence of physical state on bacterial survival during storage Biotechnol Prog 2009 25 265 270 10.1002/btpr.96 19224603

[B40] Marshall BJ Coote GG Scott WJ Some factors affecting the viability of dried bacteria during storage in vacuo Appl Microbiol 1974 27 648 652 4207761 10.1128/am.27.4.648-652.1974 PMC380110

[B41] Löster K Kannicht C Kannicht C 2-Dimensional electrophoresis: detection of Glycosylation and influence on spot pattern Post-translational Modifications of Proteins 2008 446 Totowa Nj: Humana Press 199 214 Methods in Molecular Biology™ 10.1007/978-1-60327-084-7_14 18373259

[B42] Dalle-Donne I Rossi R Giustarini D Milzani A Colombo R Protein carbonyl groups as biomarkers of oxidative stress Clin Chim Acta 2003 329 23 38 10.1016/S0009-8981(03)00003-2 12589963

[B43] Oliver JD The viable but nonculturable state in bacteria J Microbiol 2005 43 Spec No 93 100 15765062

[B44] Cabiscol E Tamarit J Ros J Oxidative stress in bacteria and protein damage by reactive oxygen species Int Microbiol 2000 3 3 8 10963327

[B45] Møller IM Rogowska-Wrzesinska A Rao RSP Protein carbonylation and metal-catalyzed protein oxidation in a cellular perspective J Proteomics 2011 74 2228 2242 10.1016/j.jprot.2011.05.004 21601020

[B46] Grune T Shringarpure R Sitte N Davies K Age-related changes in protein oxidation and proteolysis in mammalian cells J Gerontol A: Biol Med Sci 2001 56 B459 B467 10.1093/gerona/56.11.B459 11682566

[B47] Tamarit J Cabiscol E Ros J Identification of the major oxidatively damaged proteins in *Escherichia coli* cells exposed to oxidative stress J Biol Chem 1998 273 3027 3032 10.1074/jbc.273.5.3027 9446617

[B48] Cabiscol E Piulats E Echave P Herrero E Ros J Oxidative stress promotes specific protein damage in *Saccharomyces cerevisiae* J Biol Chem 2000 275 27393 27398 10852912 10.1074/jbc.M003140200

[B49] Teixeira P Castro H Kirby R Evidence of membrane lipid oxidation of spray-dried *Lactobacillus bulgaricus* during storage Lett Appl Microbiol 1996 22 34 38 10.1111/j.1472-765X.1996.tb01103.x

[B50] Williams P Winzer K Chan WC Cámara M Look who’s talking: communication and quorum sensing in the bacterial world Philos Trans Royal Soc B: Biol Sci 2007 362 1119 1134 10.1098/rstb.2007.2039 PMC2435577 17360280

[B51] Wilson DN Gupta R Mikolajka A Nierhaus KH Ribosomal proteins: Role in ribosomal functions eLS 2001 Chichester, Chichester: John Wiley & Sons, Ltd

[B52] Teixeira P Castro H Mohacsi-Farkas C Kirby R Identification of sites of injury in *Lactobacillus bulgaricus* during heat stress J Appl Microbiol 1997 83 219 226 10.1046/j.1365-2672.1997.00221.x 9281825

[B53] Fu N Chen XD Towards a maximal cell survival in convective thermal drying processes Food Res Int 2011 44 1127 1149 10.1016/j.foodres.2011.03.053

[B54] Madigan Michael T Martinko John M Stahl David A Clark David P Molecular biology of bacteria Brock biology of microorganisms 2012 13 150 235

[B55] Nagano T Kojima K Hisabori T Hayashi H Morita EH Kanamori T Miyagi T Ueda T Nishiyama Y Elongation factor G is a critical target during oxidative damage to the translation system of *Escherichia coli* J Biol Chem 2012 287 28697 28704 10.1074/jbc.M112.378067 22773838 PMC3436518

[B56] Avery SV Molecular targets of oxidative stress Biochem J 2011 434 201 210 10.1042/BJ20101695 21309749

[B57] Dalle-Donne I Aldini G Carini M Colombo R Rossi R Milzani A Protein carbonylation, cellular dysfunction, and disease progression J Cell Mol Med 2006 10 389 406 10.1111/j.1582-4934.2006.tb00407.x 16796807 PMC3933129

[B58] Maisonneuve E Fraysse L Lignon S Capron L Dukan S Carbonylated proteins are detectable only in a degradation-resistant aggregate state in *Escherichia coli* J Bacteriol 2008 190 6609 6614 10.1128/JB.00588-08 18689474 PMC2566189

[B59] Maisonneuve E Fraysse L Moinier D Dukan S Existence of abnormal protein aggregates in healthy *Escherichia coli* cells J Bacteriol 2008 190 887 893 10.1128/JB.01603-07 18039765 PMC2223551

[B60] Maisonneuve E Ezraty B Dukan S Protein aggregates: an aging factor involved in cell death J Bacteriol 2008 190 6070 6075 10.1128/JB.00736-08 18621895 PMC2546795

[B61] Cao B Liu J Qin G Tian S Oxidative stress acts on special membrane proteins to reduce the viability of *Pseudomonas syringae* pv tomato J Proteome Res 2012 11 4927 4938 10.1021/pr300446g 22928751

[B62] Desnues B Cuny C Gregori G Dukan S Aguilaniu H Nystrom T Differential oxidative damage and expression of stress defence regulons in culturable and non-culturable *Escherichia coli* cells EMBO Rep 2003 4 400 404 10.1038/sj.embor.embor799 12671690 PMC1319155

[B63] Castro HP Teixeira PM Kirby R Evidence of membrane damage in *Lactobacillus bulgaricus* following freeze drying J Appl Microbiol 1997 82 87 94 10.1111/j.1365-2672.1997.tb03301.x

[B64] Pimpo MT Seri S Study of lipid changes in freeze-dried fish during storage: I: the interaction of relative humidity and tissue lipids Boll Soc Ital Biol Sper 1992 68 735 739 1307019

[B65] Yao AA Coulibaly I Lognay G Fauconnier ML Thonart P Impact of polyunsaturated fatty acid degradation on survival and acidification activity of freeze-dried *Weissella paramesenteroides* LC11 during storage Appl Microbiol Biotechnol 2008 79 1045 1052 10.1007/s00253-008-1497-z 18461316

[B66] Oracz K El-Maarouf Bouteau H Farrant JM Cooper K Belghazi M Job C Job D Corbineau F Bailly C ROS production and protein oxidation as a novel mechanism for seed dormancy alleviation Plant J 2007 50 452 465 10.1111/j.1365-313X.2007.03063.x 17376157

[B67] Araseki M Yamamoto K Miyashita K Oxidative stability of polyunsaturated fatty acid in phosphatidylcholine liposomes Biosci Biotechnol Biochem 2002 66 2573 2577 10.1271/bbb.66.2573 12596850

[B68] Sheehy MRJ A flow-cytometric method for quantification of neurolipofuscin and comparison with existing histological and biochemical approaches Arch Gerontol Geriatr 2002 34 233 248 10.1016/S0167-4943(01)00217-5 14764326

[B69] Wislet-Gendebien S Laudet E Neirinckx V Alix P Leprince P Glejzer A Poulet C Hennuy B Sommer L Shakhova O Rogister B Mesenchymal stem cells and neural crest stem cells from adult bone marrow: characterization of their surprising similarities and differences Cell Mol Life Sci 2012 69 2593 2608 10.1007/s00018-012-0937-1 22349262 PMC11114712

